# Nanostructured TiO_2_-based gas sensors with enhanced sensitivity to reducing gases

**DOI:** 10.3762/bjnano.7.164

**Published:** 2016-11-15

**Authors:** Wojciech Maziarz, Anna Kusior, Anita Trenczek-Zajac

**Affiliations:** 1 AGH University of Science and Technology, Faculty of Computer Science, Electronics and Telecommunications, al. A. Mickiewicza 30, Krakow 30-059, Poland; 2 AGH University of Science and Technology, Faculty of Materials Science and Ceramics, al. A. Mickiewicza 30, Krakow 30-059, Poland

**Keywords:** acetone, flower-like 3D nanostructures, gas sensors, selectivity, titanium dioxide

## Abstract

2D TiO_2_ thin films and 3D flower-like TiO_2_-based nanostructures, also decorated with SnO_2_, were prepared by chemical and thermal oxidation of Ti substrates, respectively. The crystal structure, morphology and gas sensing properties of the TiO_2_-based sensing materials were investigated. 2D TiO_2_ thin films crystallized mainly in the form of rutile, while the flower-like 3D nanostructures as anatase. The sensor based on the 2D TiO_2_ showed the best performance for H_2_ detection, while the flower-like 3D nanostructures exhibited enhanced selectivity to CO(CH_3_)_2_ after sensitization by SnO_2_ nanoparticles. The sensor response time was of the order of several seconds. Their fast response, high sensitivity to selected gas species, improved selectivity and stability suggest that the SnO_2_-decorated flower-like 3D nanostructures are a promising material for application as an acetone sensor.

## Introduction

The market for resistive-type gas sensors is dominated by materials developed on the base of thin or thick layers composed of polycrystalline metal oxides. However, there is a new approach to sensor technology focusing on nanomaterials and nanostructures. It is expected that they will provide better parameters than those based on conventional materials. The nanostructures of different forms, i.e., nanowires, nanotubes, nanoflowers, have been shown to display better gas selectivity and sensitivity [1–2]. Additionally, open nanostructures facilitate the penetration of gas, and as a consequence, reduces the response time.

Titanium dioxide (TiO_2_) is effectively used in environmental and energy production applications such as dye-sensitized solar cells, photocatalytic water purification, and hydrogen generation by water splitting [3–5]. In sensor technology this n-type semiconductor is frequently considered as a promising material for gas detection applications [6]. It has excellent sensitivity and selectivity for many different gases such as H_2_ [7], NO_2_ [8], NO*_x_* [9], CO [10], NH_3_ [11], H_2_S [12], and VOCs (i.e., methanol, ethanol, propanol [13], and acetone [14]). The influence of effective surface area on the gas sensing properties of TiO_2_ thin films is also frequently observed and investigated [15].

TiO_2_ is a wide-band gap semiconductor with an extremely high resistivity extending to 10^8^ Ω·cm, however, even a small deviation from the stoichiometric composition with a surplus of titanium ions results in n-type semiconducting properties [16]. Such observations lead to the undeniable conclusions that the defect disorder and stoichiometry of the oxygen-to-titanium content ratio play a major role in the electrical properties of TiO_2_ [17].

Different ways in which TiO_2_ can be prepared include sol–gel process [18–20], flame spray synthesis [21], hydrothermal process [22], electrospinning methods [23], chemical and physical vapor deposition [24–25], and thermal, chemical, and electrochemical (anodization) oxidation [26–29]. Thermal and chemical oxidation seem to be the easiest to perform, the least expensive, and have interlaboratory reproducibility as an additional advantage. TiO_2_ is obtained mainly in form of thin film, however, recently more sophisticated nanostructures such as nanowires, nanorods, nanoplates have been exploited [23,26,30–31]. The recourse of such a direction of research results from microstructural aspects such as grain size and surface morphology, which are among the most important factors influencing the sensitivity of a gas sensor.

In comparison with spherical materials or thin layers, 3D structures exhibit much more active sites, and a complex network of canals enables gaseous reagents to penetrate inside the structure. The smaller the grains, the higher the surface-to-volume ratio for a gas sensitive material, and hence the higher the concentration of active surface oxygen adsorption centers. As a result, enhanced gas selectivity and sensitivity can be obtained. Since gas sensing properties depend on the method of synthesis and conditions applied in the preparation process, it is of crucial importance to consciously design the method of preparation.

Another way to improve sensing properties is the combination of two dissimilar materials [32]. For the past few decades, the TiO_2_/SnO_2_ coupled system has been a subject of intensive research [18,27,33]. This synergetic system modifies the electronic structure by improving electron migration from titanium dioxide to tin dioxide and promotes oxygen molecule adsorption at the surface [34]. The as-formed heterojunction (n–n type) affects the response due to the formation of the accumulation/depletion layer and increases the potential barrier at the semiconductor/gas interface [7,35]. What is even more interesting is that titanium dioxide in the form of nanoflowers, both unmodified and sensitized by SnO_2_ nanoparticles can exhibit different electrical properties under various gaseous conditions. According to the literature [26,32,36–38], there are several possible explanations for this phenomenon. The first is related to formation of titanium vacancies, *V*_Ti_, in the flower-like structure during synthesis. Upon Ti foil oxidation by H_2_O_2_, a mesoporous hierarchical structure is formed. The three-step process includes, inter alia, decomposition of titanium hydrogenperoxy gel and amorphous titania hydrate dissolution [26]. Due to these features, acceptor intrinsic defects may occur and lead to n→p transitions [36–37]. Secondly, according to Nicoloso [38], p-type conductivity is an attribute of the surface rather than the material bulk. The as-obtained pore network and fine grains of titanium dioxide nanoflowers promote high surface-to-volume ratio, which favor the p-type behavior. Finally, the presence of tin dioxide nanoparticles induce creation of intimate electrical contact at the TiO_2_/SnO_2_ interface [32], along which electron carrier transfer occurs and formation of a charge depletion layer. The discrete SnO_2_ nanoparticle coating acts as an oxygen adsorber, which donates electrons and increases carrier concentration, creating a stronger n–p heterojunction. As a result, tin oxide may become the primary conductive path, which leads to p→n transitions.

The aim of this work was to considerably improve the gas sensing properties of TiO_2_-based sensors by engineering the morphology of nanostructured TiO_2_. Layered sensor materials with different microstructures were designed and manufactured on Ti substrates. For the first time, hierarchical flower-like nanostructures obtained during the chemical oxidation process were applied in the field of gas sensor technology. The obtained sensors were analyzed as a detector of oxidizing and reducing gases. The selectivity of the nanostructures was demonstrated by a sensitivity investigation in nitrogen oxides and in the presence of acetone. The effect of surface modification on the response and recovery time was studied.

## Experimental

### Sample preparation

The sensing materials were prepared by thermal and chemical oxidation of Ti foils (*d* = 0.127 mm, 99.7%, Sigma-Aldrich). Titanium was cut into 20 × 20 mm squares and degreased in the ultrasonic cleaner with the use of acetone and isopropanol. The following samples were prepared: (A) T30 – prior to thermal treatment, the Ti foils were etched in concentrated hydrochloric acid HCl (35–38%, Avantor Performance Materials, Poland) at 55 °C for 30 min and rinsed with distilled water afterwards. The oxidation process was carried out in tubular furnace at 700 °C in air atmosphere. The air flow was controlled by a gas flow system equipped with MKS flow meters and kept at level of 80 sccm. (B) NS0 – flower-like 3D nanostructures were prepared according to the previous reports [27–28] by chemical oxidation at moderate temperature. Typically, after degreasing, the Ti foils were etched in concentrated HCl for 2 min, washed in distilled water and dried in Ar atmosphere at 100 °C. The as-prepared samples were put in 30% H_2_O_2_ at 80 °C for 30 min to obtain flower-like TiO_2_ nanostructures. To improve crystallization, the nanostructured layers were annealed at 450 °C in argon for 3 h. (C) NS1 – a soft chemistry route was used to synthesize SnO_2_ nanoparticles [39]. Flower-like TiO_2_ was immersed in a solution composed of sodium hydroxide (NaOH, 10 mM) and tin chloride pentahydrate (SnCl_4_∙5H_2_O, 0.5 mM). The mixture was stirred at 300 rpm for 24 h at room temperature. Next, the as-obtained samples were washed in deionized water and annealed at 450 °C in Ar for 1 h.

### Crystal structure and morphology

The investigation of the crystallographic structure of the materials was performed with the use of X-ray diffraction (XRD) in a grazing incidence diffraction geometry diffractometer (X’Pert Pro, Philips). Cu Kα_1_ = 0.15406 nm and Kα_2_ = 0.15444 nm radiation was applied. The phase identification was conducted with the use of HighScore Plus software and the PDF-2 database. Scanning electron microscopy (SEM) images of top- and side-view of the samples were obtained with a NOVA NANO SEM 200 (FEI) instrument.

### Gas sensitivity measurements

**Figure 1:**
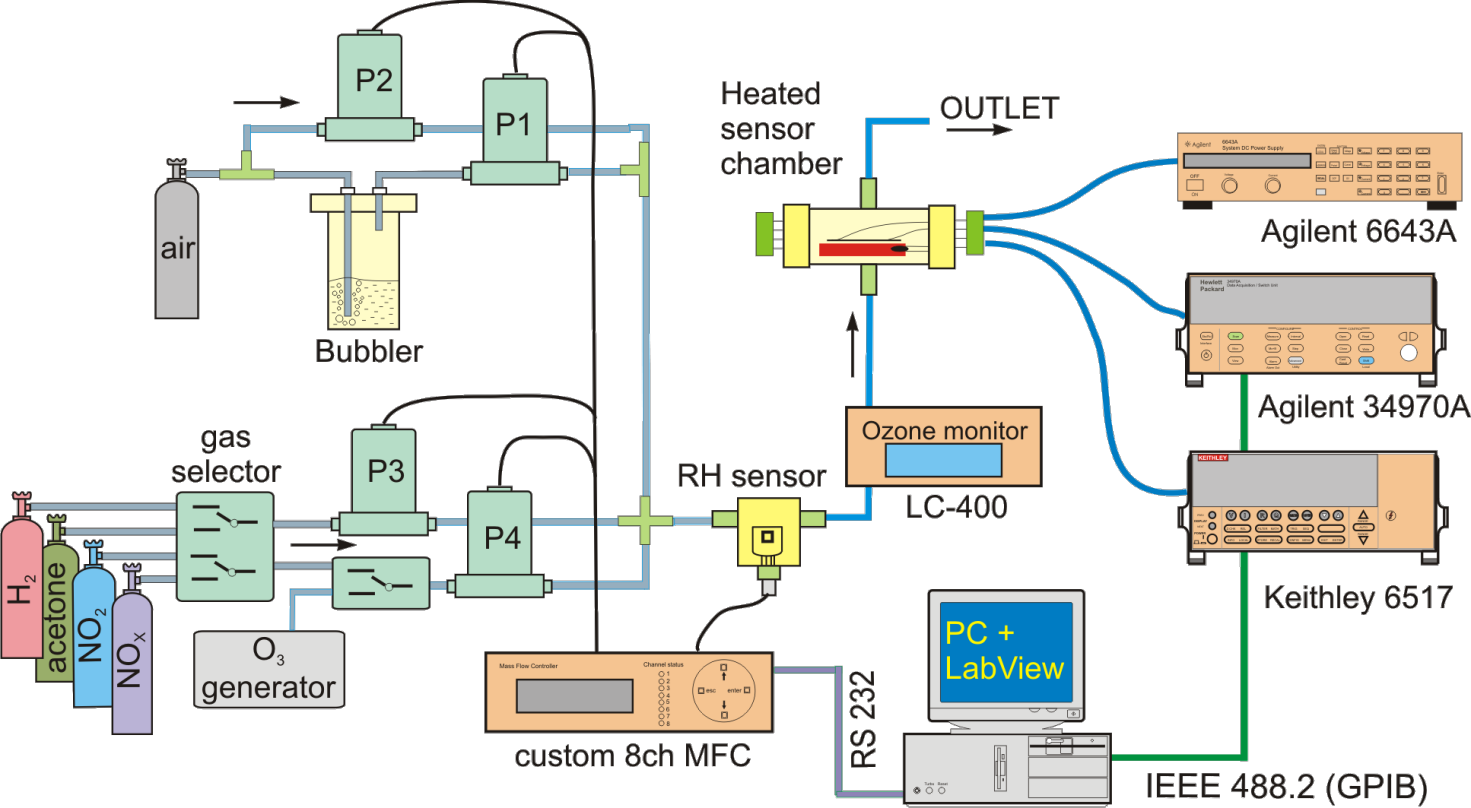
Schematic view of gas sensor measuring system.

For gas sensitivity measurements, a custom-made apparatus, presented in Figure 1, was used. The sensors were placed in a gas chamber (volume ≈30 cm^3^) on a workholder heated with an Agilent power source, model 6443A. The sensor temperature was measured with a Pt100 and Agilent 34970A digital multimeter. For the measurement of the sensor resistance, a Keithley electrometer 6517 working in constant voltage mode was used. The devices were connected to a GPIB and controlled by Agilent Technologies 82357B USB/GPIB interface from LabVIEW on a PC computer. The gas system consisted of a set of bottles, pump, bubbler (for production of humidified air), mass flow meters (type 1179 and 1459C, MKS Instruments) and custom-made mass flow and humidity controllers. The sensors were exposed to the following gases: acetone (CO(CH_3_)_2_, up to 8 ppm), nitric oxides (NO*_x_*, up to 400 ppm), hydrogen (H_2_, up to 2000 ppm), ozone (O_3_, made by a custom UV generator, up to 500 ppb), and nitrogen dioxide (NO_2_, up to 100 ppm). Additionally, the concentration of ozone was measured with an ozone monitor (LC-400, PCI-Wedeco). All gases were supplied by Air Products, Poland. The total gas flow was set to 500 sccm, and the requested gas concentration was obtained by controlling the ratio of gas to air flow rate.

Prior to measurement, the sensors were preheated in pure air atmosphere for 3–6 h under the same conditions (humidity, gas flow ratio) as used during the experiment and their response was stabilized. The preheating temperature was the starting temperature of the measurement. An example of preheating conditions for sample T30 were an air flow ratio of 500 sccm, temperature of 146 °C, relative humidity of 55%, and a stabilization time of 3 h. The preheating temperature rate was ≈3.5 °C/min. Afterwards, two different types of tests were performed: (1) the measurement of sensor response at constant gas concentration under varying temperature, and (2) the measurement of sensor response at constant temperature under varying gas concentrations. At the beginning of the measurements, the response of the sensitive layers in the presence of selected gases was determined at varying temperature. An optimum temperature range was chosen where the response to a specific gas was the highest. Then, the measurements at constant temperature with varied gas concentrations were carried out. For all the measurements, the sampling time was 2 s.

The response time, *t*_res_, and recovery time, *t*_rec_, are defined as the time required for changes in electrical resistance to occur, from the base resistance measured in air or gas (*t*_res_ and *t*_rec_, respectively) to 90% of stable signal after the gas or air introduction. On the other hand, the sensor response, *S*, was calculated as a ratio of resistance in air to resistance in gas:

[1]
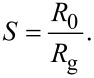


## Results and Discussion

**Figure 2:**
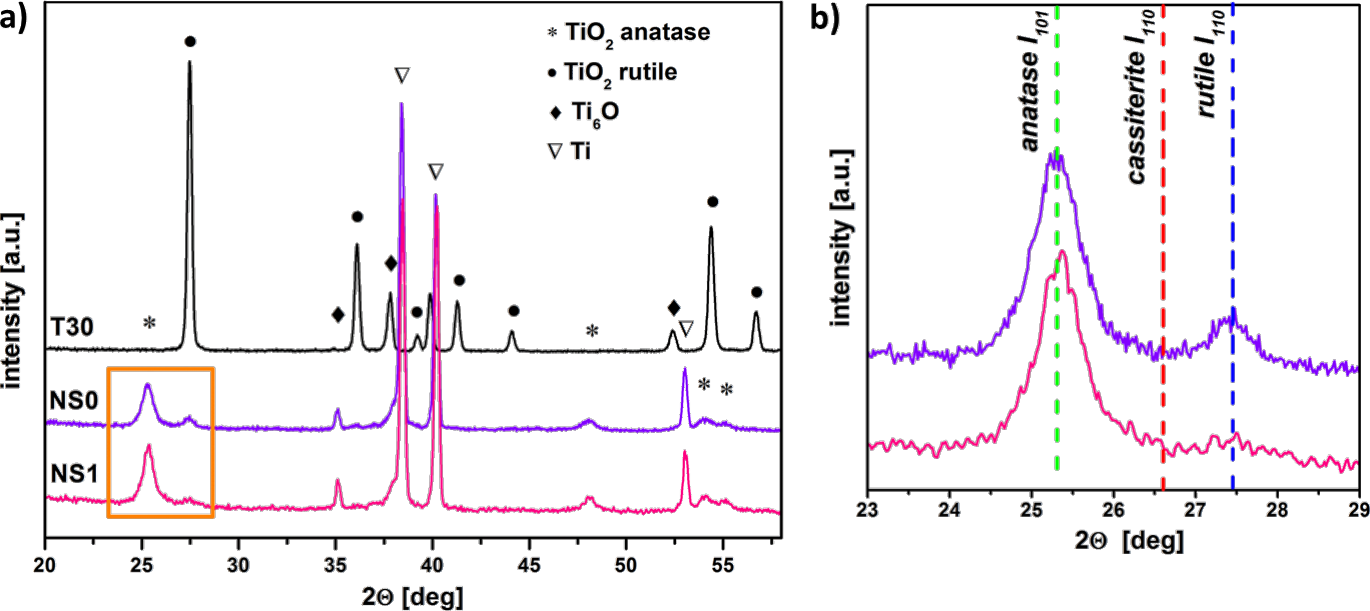
XRD patterns of TiO_2_-based sensors: a) T30 – TiO_2_ thin layer, flower-like TiO_2_ (NS0) and TiO_2_/SnO_2_ (NS1) nanostructures b) the most intense diffraction peaks of TiO_2_ (anatase, rutile) and SnO_2_ (cassiterite).

The XRD patterns of nanostructured TiO_2_ layers are demonstrated in Figure 2.

It can be observed that flower-like nanostructures crystallize in the form of anatase, with rutile as a secondary phase. Due to the extremely small tin dioxide nanoparticles, no cassiterite (SnO_2_) diffraction peaks can be distinguished, which is clearly visible in Figure 2b. This problem was discussed in our previous papers [33,39].

In the case of the T30 sensor, only reflections originating from TiO_2_ rutile phase can be observed. Characteristic peaks at the 2θ values of 34.9°, 37.8° and 52.4° appear, which are assigned to the oxygen-deficient Ti_6_O phase. The patterns related to the titanium substrate are also shown. The average crystallite size ≈40 nm for 2D nanomaterials (T30) was larger than that of the 3D structure: 11 and 13 nm for anatase and rutile, NS0 and NS1, respectively.

**Figure 3:**
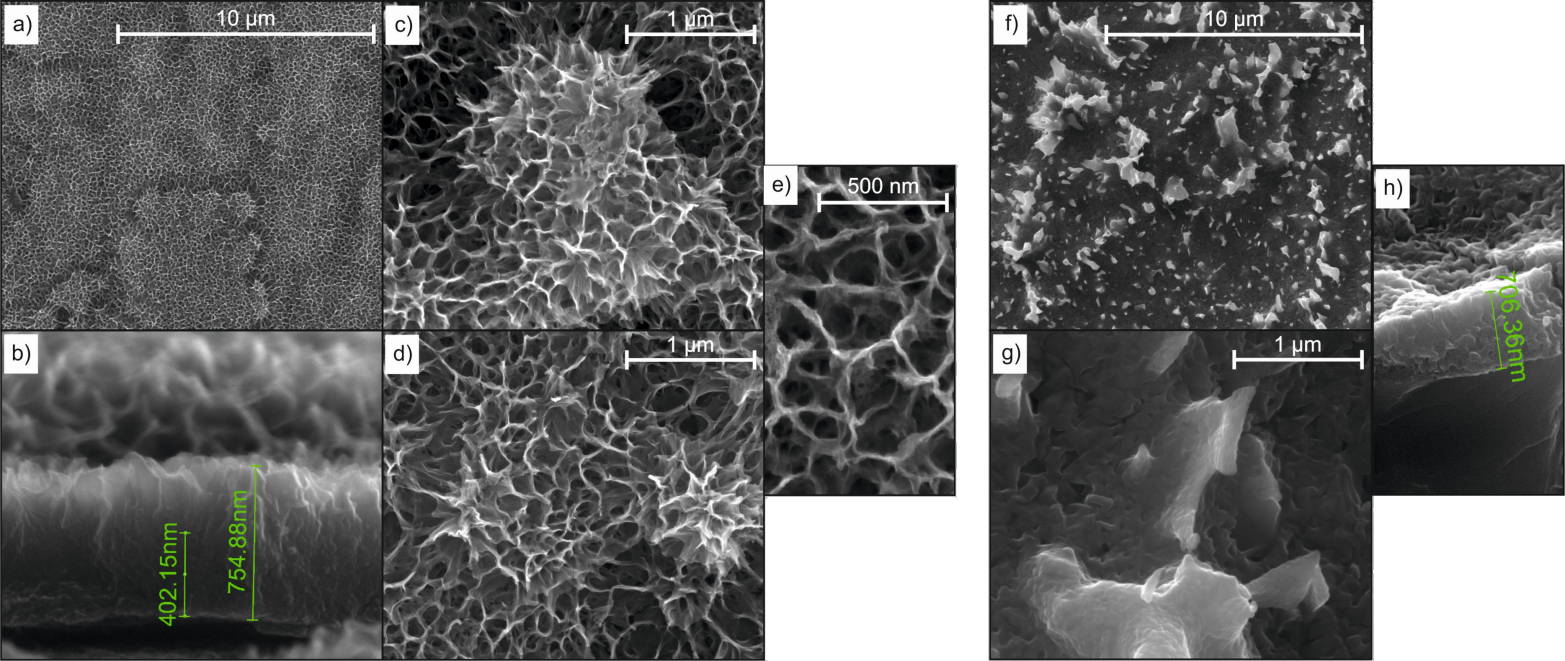
Top-views (a,c–g) and side-views (b,h) of flower-like TiO_2_ nanostructures prepared via chemical oxidation before (a–c) and after (d,e) SnO_2_ deposition, and TiO_2_-based nanostructures prepared via thermal oxidation (f–h).

The SEM images of the top- and side-views of TiO_2_-based nanostructures are presented in Figure 3. According to the side-view image (Figure 3b) and the previous analysis [27–28,39–40], the cross-section of flower-like TiO_2_ nanostructures reveals a compact layer, sponge-like form, and nanoflowers on top of the structure. The flower-like objects are also well visible on the top-views (Figure 3c,d). In the case of NS1, nanoparticles of SnO_2_ deposited on nanostructured TiO_2_, due to their size, are visible only at very high magnification (Figure 3e). The surface of T30 is also structured, which is a result of both etching and oxidation. Comparison of the side-view images (Figure 3b,h) shows that the thickness of the sensors is very similar, ≈700 nm.

**Figure 4:**
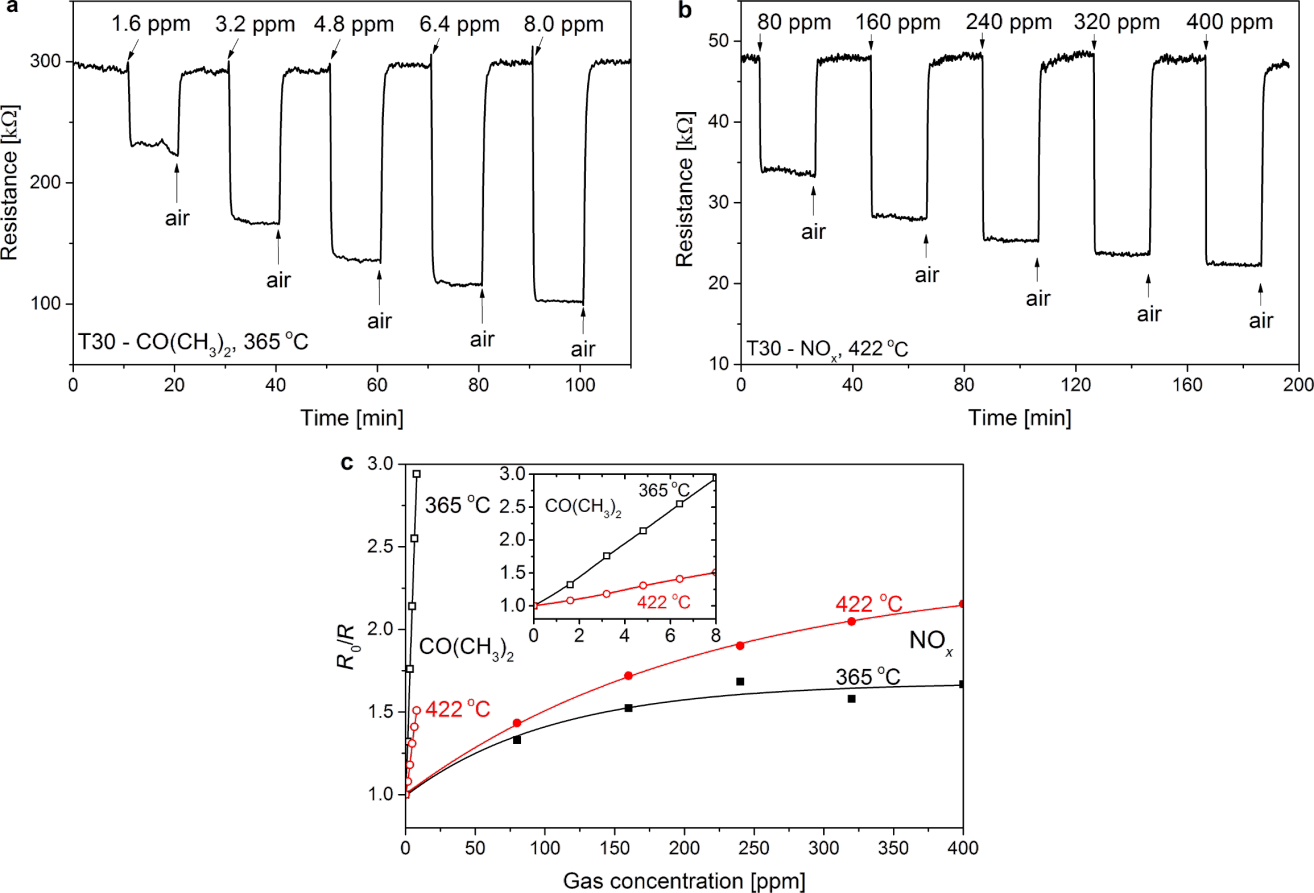
Gas sensing characteristics for the T30 sensor: dynamic changes in the electrical resistance upon exposure to (a) 1.6–8.0 ppm acetone at a constant temperature of 356 °C and (b) 80–400 ppm NO*_x_* at 422 °C, (c) values of the gas sensor response as a function of concentration at 365 °C and 422 °C.

Examples of the electrical response (Equation 1) of the T30 sensor to varying concentrations of acetone and nitric oxides, as a function of time at a constant temperature, 356 °C and 422 °C, respectively, are shown in Figure 4a,b. For both gases the sensor reacts with a large, stable and repeatable response, with step changes in the concentration range of 1.6–8.0 ppm for acetone and 80–400 ppm for nitric oxides. One can also see that there is a systematic decrease in the electrical resistance upon exposure to both gases. The response of the T30 sensor to acetone and NO*_x_* of different concentrations at the constant temperatures of 365 °C and 422 °C are presented in Figure 4c. As we can see, the sensitivity of the sensor strongly depends on temperature. According to the presented results, at lower temperature *S* is much higher for CO(CH_3_)_2_, whereas at higher temperature for NO*_x_*. Moreover, the response to NO*_x_* is nonlinear and has a tendency to flatten at higher concentrations. In the case of acetone, the characteristics are linear, which is probably due to the fact that the operating concentration range is very narrow and it is possible that application of much higher values would result in gradual flattening. This behavior is related to adsorption processes that take place on the surface of the sensor and can be described by suitable isotherms, i.e., the empirical Langmuir isotherm [41]. At a fixed surface area of the gas sensitive layer, a lower gas concentration entails a lower surface coverage with gas molecules, and subsequently a smaller number of surface reactions occur. The higher the gas concentration, the larger surface coverage, hence the number of surface reactions increases as well. This process is gradual until the saturation point on the coverage of molecules at the fixed surface area is reached. At a certain point, the increased amount of gas does not generate a sensor response as strongly as before due to the fact that the area of the uncovered surface is reduced. This process may even saturate at a fixed value in the situation when there is no further place for surface reactions to occur. Therefore, the useful sensor signal is limited.

**Figure 5:**
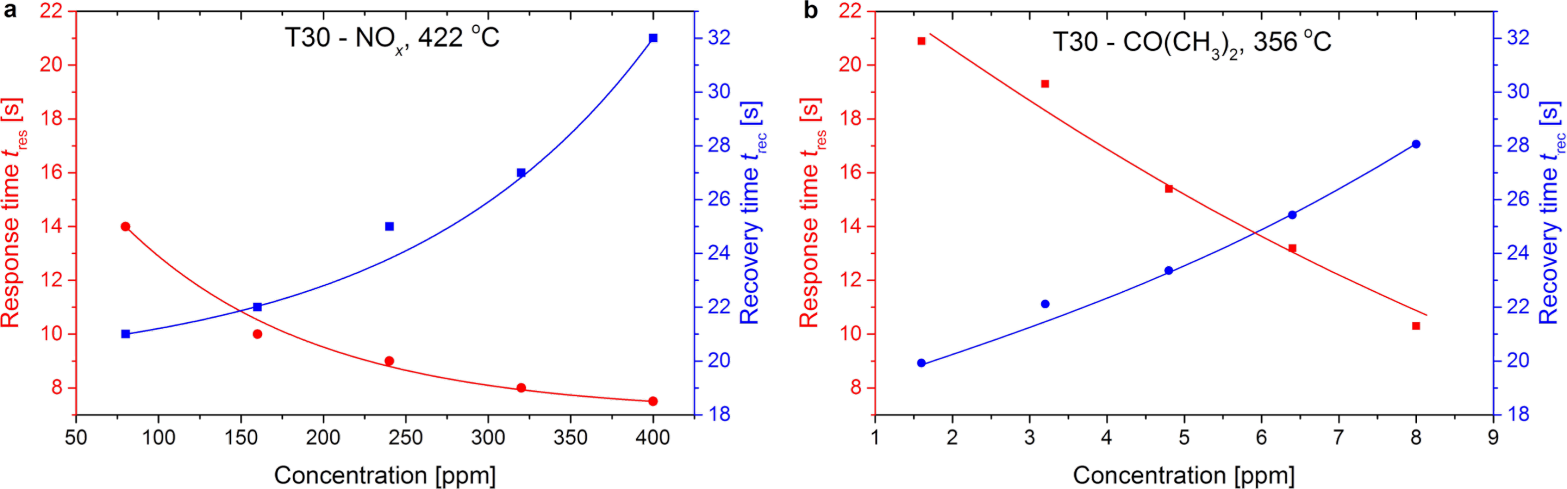
Response *t*_res_ and recovery *t*_rec_ times of the T30 sensor calculated for NO*_x_* and CO(CH_3_)_2_ at different concentrations. The temperatures were chosen based on the maximum sensitivity to selected gases.

Based on the electrical response of the T30 sensors, *t*_res_ and *t*_rec_ were calculated according to the previous definition under exposure to acetone and nitric oxides at different temperatures. In Figure 5 there is an example of *t*_res_ and *t*_rec_ as a function of changes of selected gases concentration (NO*_x_* and CO(CH_3_)_2_) at a constant temperature. As one can see, both *t*_res_ and *t*_rec_ depend on gas concentration and the temperature at which the sensor operates. Along with an increase in gas concentration, *t*_res_ becomes shorter and shorter. The change in the concentration of NO*_x_* from 80 to 400 ppm entails a decrease of *t*_res_ from 14 down to less than 8 s. On the contrary, *t*_rec_ is always longer than *t*_res_, which is also clearly seen in the case of nanostructured sensors presented in this work. When air reaches the surface of the sensor, oxygen is adsorbed on the surface and the diffusion of gas from the sensor/gas interface occurs. Desorption proceeds until all ions/particles of gas are removed from the interface. Since desorption process always takes place at temperatures higher than adsorption, longer recovery time can be easily explained. According to Basu and Basu [42] there are two ways to increase the kinetics of desorption and hence lower the recovery time, that is to raise the temperature or to inject oxygen at a higher concentration.

In comparison with the data presented in the literature [2] the response times presented on Figure 5 are significantly shorter for both TiO_2_ and TiO_2_/SnO_2_-based sensors. Considerably extended response and recovery times are observed for flower-like nanostructured sensors. This could be due to the fact that the surface of the flower-like sensor surface is very complex and more developed. Gas molecules need much more time to reach deep into the spongy surface structure of the sensor and become adsorbed.

**Figure 6:**
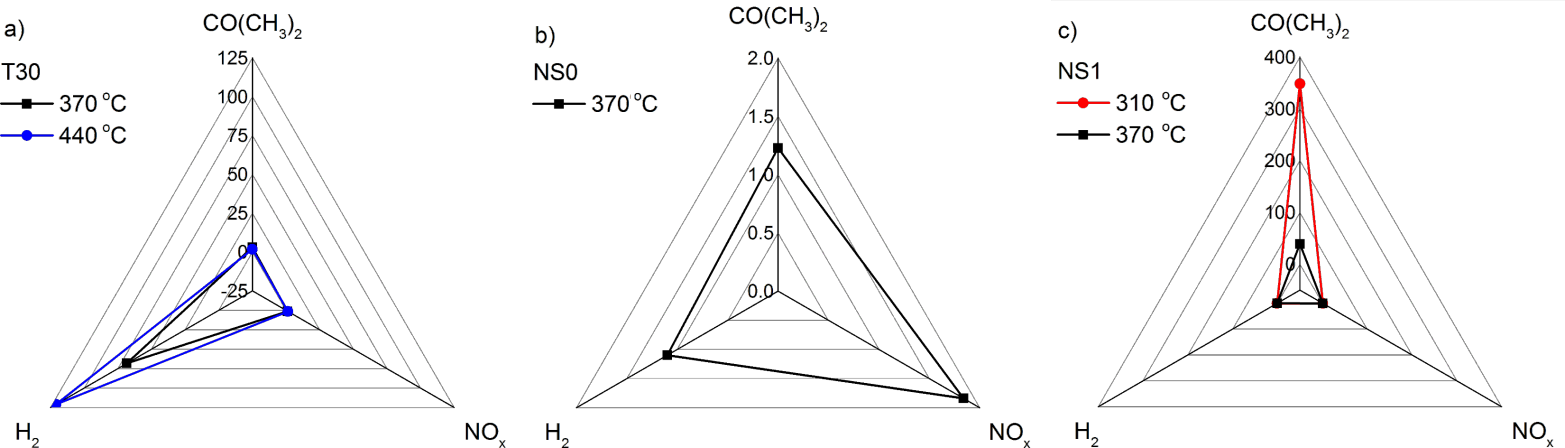
Radar plots of the response, *S*, of TiO_2_-based nanostructured sensors: (a) T30, (b) NS0 and (c) NS1 to different gases at selected temperatures.

A comparison of the response, *S*, of the TiO_2_-based nanostructured sensors to selected reducing gases at different temperatures is shown in Figure 6 in the form of radar plots. At first glance one can see that two sensors can be distinguished, in particular: T30 and NS1. The unmodified TiO_2_ layered sensor and the flower-like nanostructured TiO_2_ decorated with SnO_2_ nanoparticles are extremely selective at the presented temperatures. Namely, T30 is very sensitive to H_2_. For the flower-like TiO_2_/SnO_2_ nanostructures (NS0), a small but selective response to NO*_x_* is observed at 370 °C (*S* = 1.8). This might lead to at least two conclusions. First of all, sensitization of the flower-like TiO_2_ nanostructured sensor with SnO_2_ leads to a considerable increase in selectivity as well as increase in sensitivity to CO(CH_3_)_2_, which is according to literature [2], completely unique for both TiO_2_ and SnO_2_. Secondly, the well thought out, design engineering of the TiO_2_ microstructure allows for switching from the H_2_ highly sensitive layered sensor to the CO(CH_3_)_2_ extremely sensitive flower-like sensor. According to our previous experience in the field of photo-electrochemical properties, the creation of a SnO_2_–TiO_2_ heterojunction, whether in the form of SnO_2_ nanoparticles deposited on the flower-like TiO_2_ nanostructures, nanotubes composed of a SnO_2_–TiO_2_ solid solution, or TiO_2_ accompanied by phases from the Sn–O, significantly improves their properties [27]. A similar effect is observed when the same nanostructures are applied as gas sensors. SnO_2_ seems to catalyze the reaction to gases due to the presence of nanoparticles on the hierarchical TiO_2_ nanostructure.

**Figure 7:**
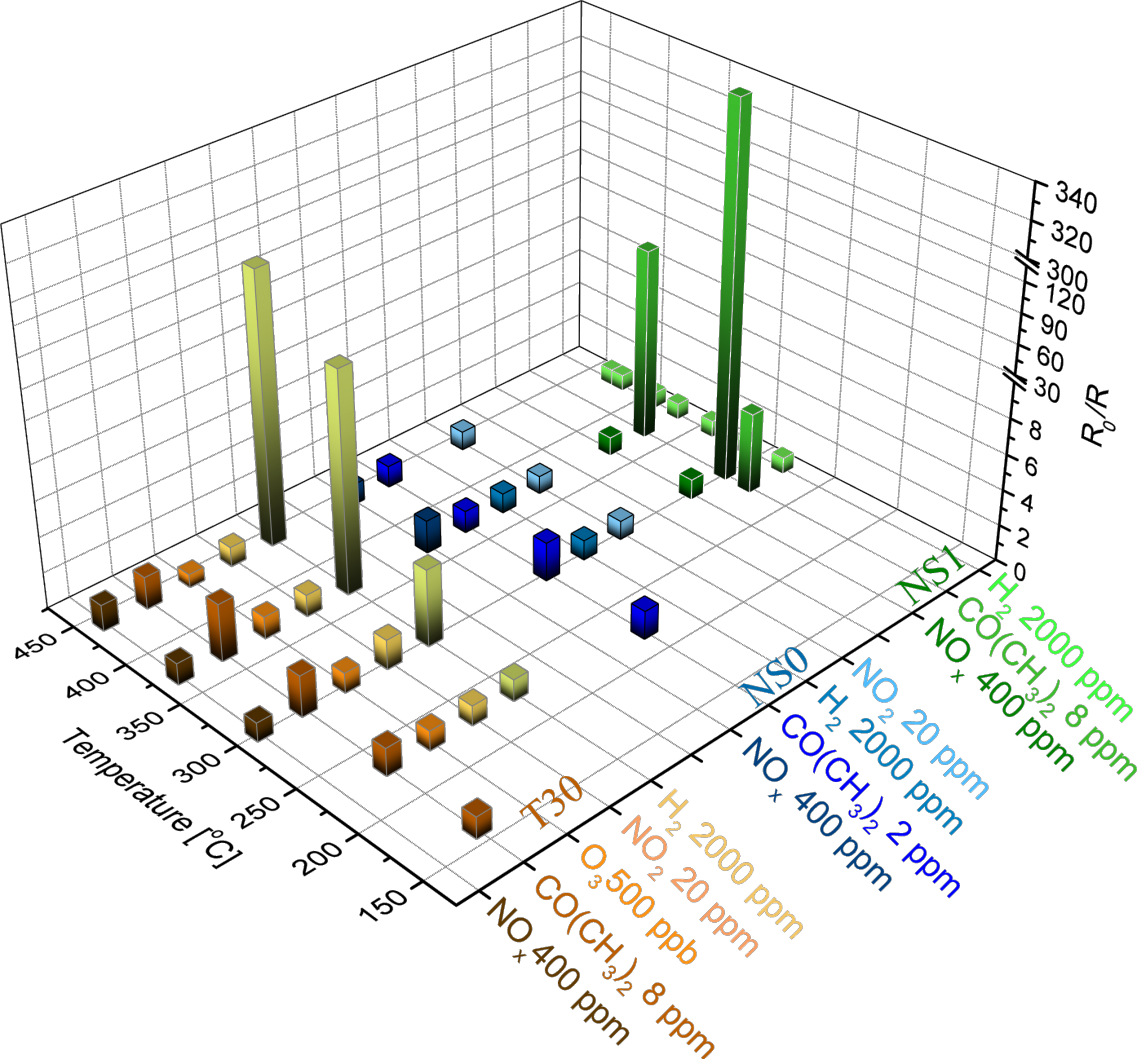
Comparison of the response of the sensors to various gases.

The response is not only dependent on the type of gas and its concentration but also on the sensor working temperature. The comparison of sensitivity vs working temperature for T30, NS0 and NS1 samples for all investigated gases is shown in Figure 7. For the T30 sample, the maximum sensitivity (*S* = 2.9) to 8 ppm of CO(CH_3_)_2_ was observed at 356 °C, while the sensitivity to 2000 ppm of H_2_ increases with temperature and reaches its maximum (*S* = 121) at 430 °C. For the NS0 sample, a very low response to the tested gases was observed. The highest observed value of *S* = 2 was for 8 ppm of CO(CH_3_)_2_ at 290 °C. The highest sensitivity to 8 ppm of acetone (*S* = 348) was observed for the NS1 sensor (flower-like 3D nanostructured sensor decorated with SnO_2_) at 308 °C.

By analyzing these results, the impact of the structure of the material and the chemical composition on the sensitivity should be considered. Firstly, morphological differences between the thin layer and flower-like structures affects the surface-to-volume ratio and thus number of active sites where gaseous molecules can be adsorbed. It is obvious that a mesoporous network system exhibits a large surface area. Moreover, the sponge-like walls (or petals) induce fast charge transport. However, lower sensitivity to various gases can be attributed to phase composition. Anatase and rutile are the most common titanium dioxide polymorphs, which exhibit different properties [43]. Anatase is a more photoactive material, while rutile, due to its effective light scattering, is widely used as a pigment. Our studies show that for nonsensitized titanium dioxide, T30 (the rutile-based sample) is a much better sensor than NS0 (the anatase-based material).

According to Wang et al. [44], the enhanced sensitivity of a flower-like form doped with SnO_2_ nanoparticles can be attributed to gas sensing properties of tin dioxide. This archetypical sensor material affects the formation of the depletion layer at the surface. As a consequence, electron charge carriers have to overcome a high potential barrier formed at the semiconductor/gas interface. However, due to their small dimensionality and good dispersion on TiO_2_ substrates, a large number of active sites are created and depletion layer thickness decreases. Our previously proposed sensing mechanism [7] for this semiconductor coupled system remains in good correlation with the obtained results.

## Conclusion

In this work, the crystal structure, morphology and gas sensing properties of the TiO_2_ nanostructures were investigated. The samples were prepared by chemical and thermal oxidation of Ti substrates. The physicochemical properties of nanostructures with different microstructure were investigated: thin layer 2D and flower-like 3D nanostructures as well as 3D structures decorated with SnO_2_. It was found that crystal structure and morphology of the sensors affect the material selectivity. The sensor based on a thin layer of titanium dioxide (rutile) exhibited the best performance for H_2_ detection. On the contrary, the flower-like TiO_2_ nanostructured sensor (anatase) not only showed almost no response to H_2_ but also demonstrated moderate response to NO*_x_*. Despite the extensive pore network and possible number of active sites, the flower-like TiO_2_ showed the lowest sensor activity. However, it was also found that modification of the flower-like 3D nanostructures by decorating with SnO_2_ nanoparticles allows for a sensor with a very high response to CO(CH_3_)_2_. It is also worth emphasizing that the increasing gas concentration leads to a decrease of the response time down to several seconds. Due to the complex mesoporous structure, gas desorption was difficult, which influenced recovery time of the flower-like 3D samples. This work suggests that SnO_2_-decorated flower-like TiO_2_ 3D nanostructures are a promising material for application as a CO(CH_3_)_2_ sensor.
